# Evaluating nivolumab plus gemcitabine–cisplatin’s cost-effectiveness for aUC in China

**DOI:** 10.3389/fphar.2024.1382342

**Published:** 2024-11-05

**Authors:** Kehui Meng, Heng Xiang, Meiyu Wu, Ouyang Xie, Andong Li, Chongqing Tan, Xiaomin Wan

**Affiliations:** Department of Pharmacy, Second Xiangya Hospital, Central South University, Changsha, China

**Keywords:** advanced urothelial carcinoma, nivolumab, cost-effectiveness, quality adjusted life years, immunotherapy, Pharmacoeconomics

## Abstract

**Aims:**

Assessing the cost-effectiveness of Nivolumab with Gemcitabine–Cisplatin for Advanced Urothelial Carcinoma (aUC) treatment from the perspective of Chinese payers.

**Methods:**

A Markov model assessed economic outcomes, estimating health outcomes in quality-adjusted life years (QALYs). One-way and probabilistic sensitivity analyses were conducted to assess the impact of uncertainties on the results.

**Results:**

The base-case analysis showed Nivolumab plus Gemcitabine–Cisplatin yielded 0.59 QALYs at an extra cost of $78,780.61, leading to an incremental cost-effectiveness ratios (ICER) of $133,526.46/QALY. One-way sensitivity analysis highlighted Nivolumab’s cost as the key factor, while probabilistic sensitivity analysis showed a 0% chance of cost-effectiveness for Nivolumab plus Gemcitabine–Cisplatin in aUC treatment.

**Conclusion:**

Nivolumab plus Gemcitabine–Cisplatin is not cost-effective in the treatment of aUC.

## 1 Introduction

Bladder cancer ranks among the top ten most common cancers worldwide (van Hoogstraten et al., 2023), affecting the urinary tract’s inner lining from the renal pelvis to the distal urethra (Cassell et al., 2021). Nearly 1.65 million people are affected by bladder cancer globally, leading to approximately 550,000 new cases each year (Richters et al., 2020). This represents 3% of all newly identified cancer cases and accounts for 2.1% of deaths caused by cancer. Urothelial carcinoma (UC) is the primary histological type, comprising over 90% of all cases (Hepp et al., 2021), thus imposing a significant burden on healthcare systems.

Cisplatin has played a pivotal role in the treatment of UC since the 1970s, demonstrating response rates ranging from 30% to 70% in initial reports for advanced UC treated with cisplatin-containing regimens (Sternberg et al., 1977; Yagoda, 1977; Sternberg et al., 1985). Over the last decade, the treatment landscape for aUC has witnessed significant transformations (Santini et al., 2023). Immunotherapy, characterized by an improved safety profile and more prolonged responses compared to platinum-based chemotherapy (Ferrer-Mileo et al., 2020; Giannatempo et al., 2023; Miyake et al., 2023), is reshaping patient care. Immune checkpoint inhibition (CPI) stands at the forefront of innovations in patient management strategies (Martini et al., 2022).

Recently, the results of the phase III trial CheckMate 901 were reported. The study’s objective was to assess the effectiveness and safety of combining nivolumab with gemcitabine-cisplatin versus using gemcitabine-cisplatin alone for the initial treatment of patients facing advanced urothelial carcinoma that is either inoperable or metastatic (van der Heijden et al., 2023).

In this clinical trial, the nivolumab-combination therapy demonstrated a significantly extended overall survival. The median overall survival (OS) reached 21.7 months (95% CI, 18.6–26.4) in the nivolumab combination group, surpassing the 18.9 months (95% CI, 14.7–22.4) observed in the gemcitabine–cisplatin group (van der Heijden et al., 2023). Progression-free survival (PFS) also exhibited a significant increase in the nivolumab-combination group, with a median progression-free survival of 7.9 months (95% CI, 7.6–9.5) compared to 7.6 months (95% CI, 6.1–7.8) in the gemcitabine–cisplatin group (van der Heijden et al., 2023).

The combination of nivolumab and gemcitabine-cisplatin presents itself as a promising regimen for treating aUC. Yet, the cost-effectiveness of nivolumab in the context of aUC treatment decisions remains uncertain in China. This study endeavors to assess the cost-effectiveness of nivolumab in conjunction with gemcitabine-cisplatin as a first-line therapy for aUC, adopting the perspective of Chinese payers.

## 2 Material and methods

### 2.1 Decision model

The study focused on participants over the age of 18 diagnosed through histology with inoperable or spreading urothelial cancer affecting areas such as the renal pelvis, ureter, bladder, or urethra, following the guidelines set by the CheckMate 901 trial (van der Heijden et al., 2023).

**FIGURE 1:**
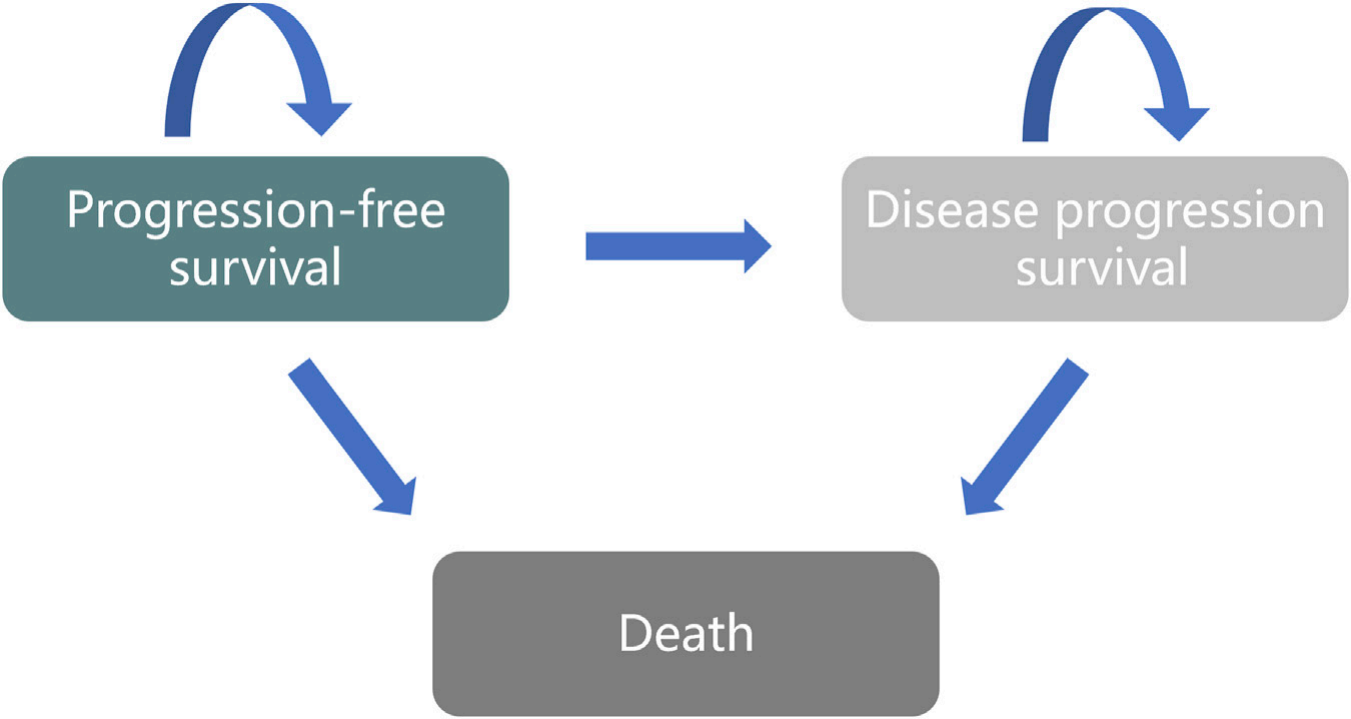
State Transition Diagram. The three circles represent the three main health states. Patients can transition from “Progression-Free Survival” to either “Disease Progression Survival” or “Death.”

A Markov model was developed to evaluate the cost and effectiveness of initial treatment options for patients, which showcases three distinct health states: PFS, Disease Progression (PD), and Death (Figure 1).

The analysis compared the outcomes of two first-line treatments: nivolumab combined with gemcitabine-cisplatin and gemcitabine-cisplatin on its own. Treatment with platinum-based chemotherapy was halted after six cycles for both groups. For those receiving the combination therapy, nivolumab treatment was extended until the cancer progressed. Following progression, individuals in both cohorts were administered a second-line therapy. In both groups, the second-line treatment involved the use of gemcitabine plus cisplatin. According to the Bladder Cancer Treatment Guidelines (2022) (Xing et al., 2022), the preferred option for patients who fail platinum-based chemotherapy is immune checkpoint inhibitors, and for those who fail immune checkpoint inhibitors, the preferred option is platinum-based chemotherapy. However, this approach would render the model’s results incomparable. Hence, the analysis proceeds under the assumption that platinum-based chemotherapy is administered as the secondary line of treatment to all participants.

The model employed a 1-week Markov cycle and a lifetime horizon of 10 years. Direct model care costs were exclusively considered (Table 1). In this analysis, both cost and utility values were subjected to discounting with an annual rate of 5%, in alignment with the China Guidelines for Pharmacoeconomic Evaluations (2020) (Liu et al., 2020). Additionally, the model incorporated half-cycle correction.

**TABLE 1:** Model parameters: baseline values, ranges, and distributions for sensitivity analysis.

Parameter	Base case	Range	Distribution	Source
Cost input, US$				
Nivolumab cost	1249.072/100 mg	999.2576–1498.886	Gamma	Kangzhou Big Data (2023)
Gemcitabine cost	5.596/0.2 g	4.4768–6.7152	Gamma	Kangzhou Big Data (2023)
Cisplatin cost	1.059/10 mg	0.8472–1.2708	Gamma	Kangzhou Big Data (2023)
Administration per unit	41	30.75–51.25	Gamma	Shu et al. (2023)
Terminal care in end-of-life	278.21	222.57–333.85	Gamma	Shu et al. (2023)
AE cost, US$				
Anemia	500.78	445.76–545.54	Gamma	Yang et al. (2023)
Neutropenia	434.57	0–1290.65	Gamma	Yang et al. (2023)
Decrease Neutrophil count	534.4	427.52–641.28	Gamma	Zhang et al. (2023)
Decreased white-cell count	622.5231	498.02–747.03	Gamma	Jiang et al. (2023)
Utility				
PFS	0.8	0.77–0.82	Beta	Qin et al. (2021)
PD	0.71	0.57–0.85	Beta	Qin et al. (2021)

PFS: progression-free survival; PD: Disease Progression; AE: adverse effect; US$: US dollars.

The study aimed to measure key outcomes including overall expenses, life years (LYs), QALYs, and ICERs. The ICER threshold, reflecting the willingness to pay (WTP), was set at three times the *per capita* GDP of China for the year 2022 (Liu et al., 2020), equating to $38,223 (Statistics, 2023).

The Markov model construction and supplementary statistical analyses were executed using R software (version 4.3.1; http://www.r-project.org).

### 2.2 Model progression and survival estimates

Unlike partitioned survival model, where the proportion of cohorts in each state was estimated directly from the area under the associated survival curve, Markov model evaluated the number of individuals in each state using transition probabilities. We estimated transition probabilities between different health states based primarily on the CheckMate 901 trial (van der Heijden et al., 2023). Data points for the PFS and OS were extracted utilizing the WebPlotDigitizer tool (https://apps.automeris.io/wpd/index.zh_CN.html). We reconstructed individual patient-level data using the IPDfromKM software package available online (version 0.1.10) and fitted these data with several parametric distributions (Exponential, Weibull, Lognormal, Log-logistic, and Gompertz). Guided by the Akaike information criterion (AIC), the analysis found that log-logistic models were most suitable for describing the survival curves, including OS for both treatment arms and PFS for the gemcitabine–cisplatin arm. Lognormal models best captured the PFS for the nivolumab-combination group (Supplementary Material 1, Supplementary Table S1). Based on the selected parameter model, we then used the equations described below to calculate the transition probability from one cycle to the next (Briggs et al., 2006; Zhou, 2018);

$$tp\left(t_{u}\right)=1-S\left(t\right)/S\left(t-u\right)$$

Additionally, age-specific mortality rates from other causes were incorporated into the model, sourced from the CHINA POPULATION CENSUS YEARBOOK 2020 (Supplementary Material 1, Supplementary Table S2) (Census, 2023).

### 2.3 Cost estimates

The analysis focused exclusively on direct medical expenses: encompassing drug costs, the expenses related to grade≥3 adverse events (AEs): Anemia, Decreased white-cell count, Decreased neutrophil count and Neutropenia, and terminal care costs (Table 1). Drug costs per cycle for the first-line treatment were computed based on the following dosages. Nivolumab (at a dose of 360 mg) in combination with gemcitabine–cisplatin every 3 weeks for a maximum of six cycles. Subsequently, nivolumab (at a dose of 480 mg) was administered every 4 weeks starting 3 weeks after the last combination therapy until disease progression or a maximum of 2 years. The other group received gemcitabine–cisplatin alone every 3 weeks for up to six cycles. The gemcitabine–cisplatin chemotherapy protocol entailed administering Gemcitabine 1,000 mg/m^2^ on days 1 and 8 of the cycle and Cisplatin 70 mg/m^2^ on day 1 (van der Heijden et al., 2023).

For calculating dosage, the study assumed an average body weight of 65 kg and a height of 1.65 m (Yang et al., 2023). All financial figures are presented in US dollars, with the exchange rate set at $1 to ¥7.1470.

### 2.4 Utility estimates

Overall QALYs were calculated by adjusting survival time for health-related quality of life. Utilities for PFS and PD states were set at 0.80 and 0.71 (Qin et al., 2021), respectively (Table 1).

### 2.5 Analysis

During the base case analysis, the ICER baseline value was determined. Further, subgroup analyses were performed based on CheckMate 901 trial outcomes. Specifically, for patients exhibiting a PD-L1 expression of 1% or higher, the nivolumab combination was preferred over gemcitabine–cisplatin alone in terms of both OS and PFS (van der Heijden et al., 2023), as indicated by favorable hazard ratios. This led to conducting a targeted subgroup analysis.

To verify the base case findings’ stability, both one-way and probabilistic sensitivity analyses were executed. The one-way sensitivity analysis adjusted every parameter by ±20% of the base value or within their 95% confidence intervals. Meanwhile, the probabilistic sensitivity analysis employed a Monte Carlo simulation with 5,000 runs, randomly selecting all parameters from their distributions simultaneously (Table 1).

## 3 Results

### 3.1 Base case and subgroup analysis

We estimated the estimated probabilities of events (Supplementary Material 2, Supplementary Table S5) and the transition probabilities between states in the model (Supplementary Material 2, Supplementary Table S6) based on data from the CheckMate 901 trial.

In the base case analysis, the combination of nivolumab treatment resulted in a gain of 0.59 QALYs at an additional expense of $78,780.61, leading to a ICER of $133,526.46 per QALY ($103,658.70 per LY) in comparison to the standard gemcitabine–cisplatin regimen (Table 2).

**TABLE 2:** Base case results.

Results	Nivolumab-combination	Gemcitabine–cisplatin	Incremental
LYs	3.70	2.94	0.76
QALYs	2.77	2.18	0.59
Total cost, US$	84,617.97	5837.36	78,780.61
ICER, US$			
Per LY			103,658.70
Per QALY			133,526.46

LY, life year; QALY, quality-adjusted life year; ICER, incremental cost-effectiveness ratio; US$, US dollars.

In the subgroup analysis, for patients exhibiting a PD-L1 expression level of 1% or higher, an increment of 0.85 QALYs was observed, with the ICER valued at $97,905.48 per QALY (Supplementary Material 1, Supplementary Table S3).

### 3.2 Sensitivity analysis

**FIGURE 2:**
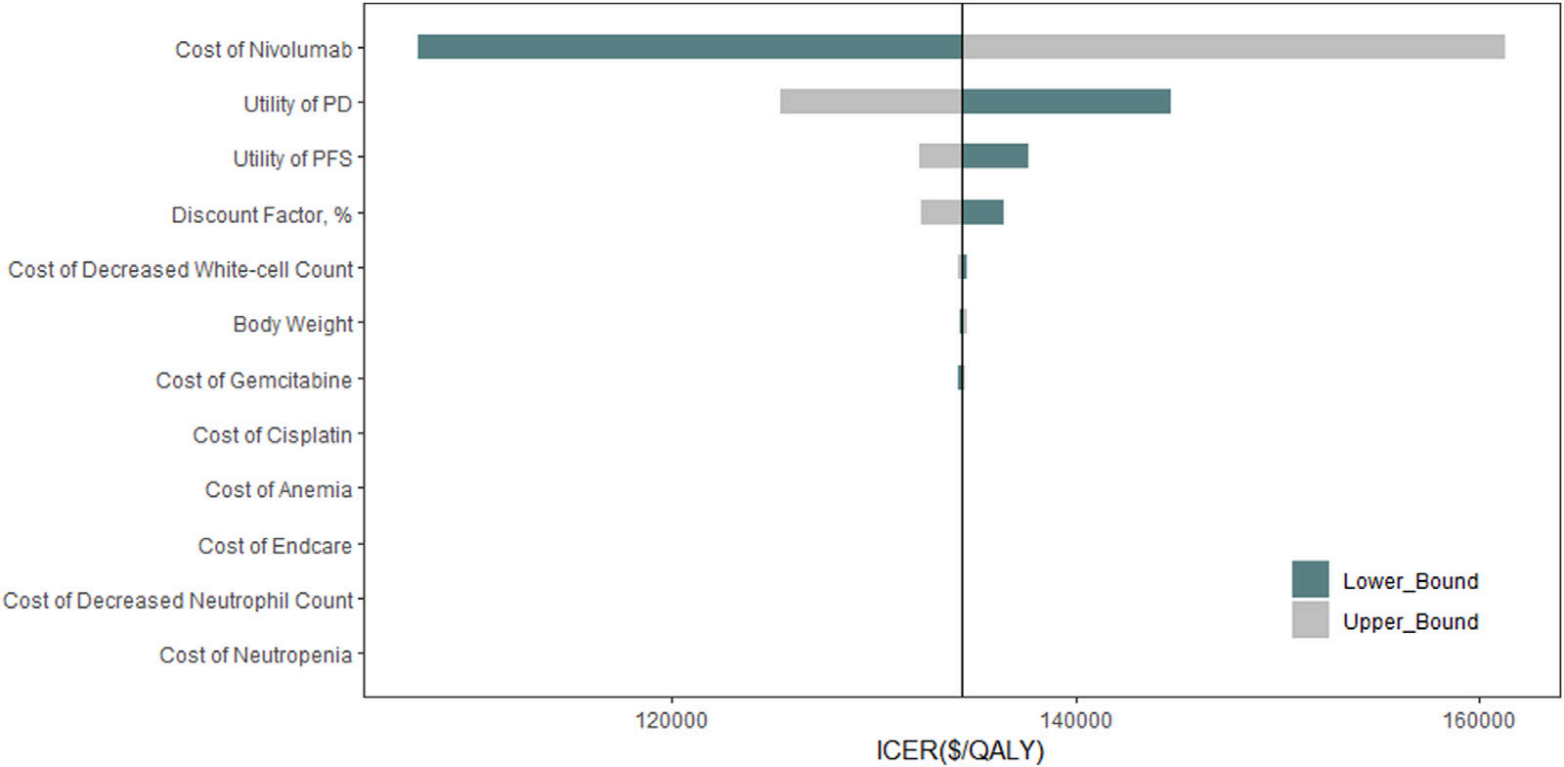
Tornado Diagram. This diagram shows the impact of different individual model inputs on the cost-effectiveness of Nivolumab combined with chemotherapy in treating aUC. ICER: Incremental Cost-Effectiveness Ratio; QALY: quality-adjusted life year; PFS: progression-free survival; PD: Disease Progression.

**FIGURE 3:**
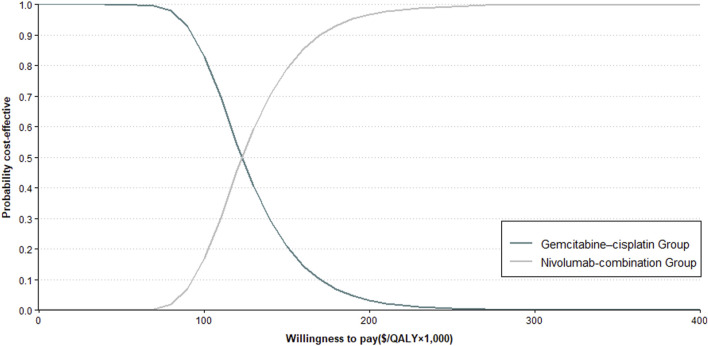
Cost-Effectiveness Acceptability Curve. This figure illustrates the probabilistic sensitivity analysis outcomes (refer to Methods for details) comparing Nivolumab plus Gemcitabine–Cisplatin against gemcitabine–cisplatin alone for aUC.

The sensitivity analysis revealed the cycle cost of nivolumab, along with the utility values for PD and PFS, significantly impacts the ICER (Figure 2). The probabilistic analysis demonstrated no cost-effectiveness for the nivolumab-combination at the set WTP threshold (Figure 3).

A reduction of 72.1% in nivolumab’s cycle cost is needed to achieve an ICER under the specified threshold, as detailed in the threshold analysis (Supplementary Material 1, Supplementary Table S4).

## 4 Discussion

Our findings suggest that in the context of aUC, the combination of Nivolumab with platinum-based chemotherapy does not present a cost-effective alternative when measured against the standard treatment of gemcitabine–cisplatin chemotherapy. The ICER, valued at $133,526.46 per QALY, substantially surpasses the WTP threshold set at $38,223 per QALY. This outcome is consistent with findings from a previous UK-based analysis that also evaluated the clinical and cost-effectiveness of Nivolumab against other treatments like docetaxel, paclitaxel, and best supportive care for aUC (Grimm et al., 2019). However, the costs of treating urothelial carcinoma vary significantly across countries. For example, the annual drug costs in Italy are twice those in France and the United Kingdom (Contieri et al., 2024). Therefore, the cost-effectiveness of treating aUC in different countries still needs to be analyzed in conjunction with local conditions.

However, the high cost of Nivolumab does not mean that patients must resort to less effective chemotherapy. The 2022 guidelines from China’s National Cancer Center endorse gemcitabine plus cisplatin as the preferred initial therapy for metastatic bladder urothelial carcinoma patients who can endure cisplatin (Xing et al., 2022). Despite platinum-based chemotherapy being effective in over 40% of cases, with a median survival period of approximately 15 months, long-term benefits are rare (von der Maase et al., 2005; von der Maase et al., 2023; Yu et al., 2023). This highlights the need for alternative treatments with more sustainable outcomes. In these guidelines, Nivolumab, as a form of immunotherapy, has been used alone for the treatment of UC (Xing et al., 2022). Yet, the combination of nivolumab with chemotherapy in various cancers, compared to chemotherapy alone, has been found to bring additional benefits in terms of PFS and OS (Janjigian et al., 2021; Doki et al., 2022). The CheckMate 901 trial specifically highlights the advantages of integrating PD-1 inhibition with platinum-based chemotherapy regimens.

The introduction of PD-1/PD-L1 inhibitors has marked a notable progression in the treatment of aUC, yet the optimal application of these therapies in everyday clinical settings remains under exploration (Hussain et al., 2018). Indeed, the CheckMate 901 study revealed that for individuals battling metastatic urothelial carcinoma, the integration of Nivolumab with gemcitabine-cisplatin not only enhanced survival rates but also elicited profound and enduring responses in a significant fraction of the patient population (van der Heijden et al., 2023). The combined use of Nivolumab and gemcitabine-cisplatin has shown a safety profile consistent with prior trials involving patients with urothelial carcinoma, where instances of treatment-related mortality were notably rare. (Sharma et al., 2017; Bajorin et al., 2021; von der Maase et al., 2023).

While innovation enhances treatment outcomes, it also escalates healthcare expenses (DiMasi et al., 2003), and current data on the use of Nivolumab, particularly in conjunction with platinum-based chemotherapy as an initial treatment for aUC, remains sparse. This study is pioneering in conducting a cost-effectiveness analysis from the perspective of Chinese payers on the use of Nivolumab with platinum-based chemotherapy for aUC. Given the findings from our analysis, incorporating Nivolumab with platinum-based chemotherapy does not emerge as the superior initial treatment strategy for patients with aUC when evaluated from a value-based perspective.

Cost-effectiveness analysis can aid in determining drug pricing, reflecting the benefits of the drug to patients and the healthcare system (Goldstein et al., 2015). Adjusting the cost of Nivolumab and its combination with platinum-based chemotherapy is essential for enhancing the affordability and cost-effectiveness of this treatment regimen.

The one-way sensitivity analysis highlighted that the cost per cycle for Nivolumab significantly affects the overall cost-effectiveness outcome. However, even with a 50% reduction in the cycle cost of Nivolumab, the ICER remains above the cost-effectiveness threshold of $38,223 per QALY. Reducing the cost of Nivolumab’s treatment cycle by 72% would align its use as a first-line treatment for aUC patients with the WTP threshold of $38,223 per QALY, thereby rendering it cost-effective.

To ensure that the combination of Nivolumab with platinum-based chemotherapy for aUC is both cost-effective and affordable, it is essential to reduce the price of Nivolumab. Currently, four PD-1 inhibitors are included in health insurance, with Tislelizumab and Toripalimab indicated for urothelial carcinoma (National Healthcare Security Administration, 2023). Tislelizumab was included in the insurance plan on 1 March 2021, with a pre-insurance price of $1,495.45 for 100 mg, now reduced to just $175.39, representing an 88% price drop (Kangzhou Big Data, 2023). The price for 240 mg of Toripalimab before entering the insurance was $1,007.42, and the current insurance price is only $263.73 marking a 73% reduction (Kangzhou Big Data, 2023). Research indicates that a 70% reduction in the cost of Nivolumab would make it cost-effective; although this is a significant price drop, it is not impossible. While there are no head-to-head clinical trials currently available comparing Nivolumab with Tislelizumab or Toripalimab in urothelial carcinoma patients, if Nivolumab successfully gains insurance coverage, more patients with urothelial carcinoma could benefit from it.

This research acknowledges certain limitations. Firstly, the utility values utilized in the model, derived from earlier studies, might not accurately mirror the utility estimates obtained in the CheckMate 901 trial. Secondly, for second-line treatment, we assumed the use of gemcitabine plus cisplatin. Although according to the one-way sensitivity analysis, the cost of second-line treatment has minimal impact on the model’s outcome. Third, our model fundamentally relies on the validity and generalizability of the CheckMate 901 trial. Long-term survival data were extrapolated by applying parameter distributions to the short-term survival data from the trial. Despite testing the fit of parameter distributions based on the AIC, the extrapolated survival curves still possess inherent uncertainties. The long-term benefits of Nivolumab combined with platinum-based chemotherapy remain insufficiently evidenced. Lastly, the data from clinical trials may not accurately reflect real-world conditions, where the situation is complex and variable. Patients may have multiple comorbidities, and adherence and insurance factors may differ from those preset in clinical trials. Therefore, the emergence of new evidence or real-world data regarding the use of Nivolumab as a primary treatment for aUC would prompt a necessary update to this study’s findings and conclusions. Therefore, conclusions should be interpreted and referenced with caution.

## 5 Conclusion

Our research shows that for Chinese payers, using Nivolumab in combination with platinum-based chemotherapy does not offer a cost-effective solution for aUC patients, especially when considering a WTP benchmark of $38,223 per QALY. Despite Nivolumab’s ability to improve PFS and OS without a significant uptick in AEs, it still does not emerge as the preferred initial therapy over conventional platinum-based treatments. Lowering Nivolumab’s cost might make it a more economically viable option for treating aUC.

## Data Availability

The original contributions presented in the study are included in the article/Supplementary Material, further inquiries can be directed to the corresponding authors.
