# Supplementary material for: Evaluating nivolumab plus gemcitabine–cisplatin’s cost-effectiveness for aUC in China
Source: Front Pharmacol. 2024 Nov 5;15:1382342. doi: 10.3389/fphar.2024.1382342 (PMC11573544; doi:10.3389/fphar.2024.1382342)
Supplement: Supplementary file 2 [file DataSheet1.docx]

Table S1. AIC values for each parametric survival models

| Parametric survival models | Nivolumab-combination group | Gemcitabine–cisplatin group |
| --- | --- | --- |
| Overall survival |  |  |
| Exponential | 1564.175 | 1652.896 |
| Weibull | 1563.973 | 1650.327 |
| Log-normal | 1553.556 | 1662.508 |
| Log-logistic | 1552.930 | 1647.389 |
| Gompertz | 1565.909 | 1654.260 |
| Progression-free survival |  |  |
| Exponential | 1609.238 | 1278.408 |
| Weibull | 1611.113 | 1267.480 |
| Log-normal | 1556.359 | 1247.733 |
| Log-logistic | 1557.171 | 1239.094 |
| Gompertz | 1589.254 | 1280.408 |

Abbreviations: AIC, Akaike information criterion; Nivolumab-combination group, Nivolumab plus platinum-based chemotherapy; Gemcitabine–cisplatin, Gemcitabine–cisplatin chemotherapy.

Table S2. Age-specific mortality rate

| **Age** | **Mortality Rate** |
| --- | --- |
| 65 | 0.00949 |
| 66 | 0.01062 |
| 67 | 0.01168 |
| 68 | 0.01335 |
| 69 | 0.01455 |
| 70 | 0.01619 |
| 71 | 0.01869 |
| 72 | 0.02056 |
| 73 | 0.02279 |
| 74 | 0.02529 |
| 75 | 0.02889 |
| 76 | 0.03165 |
| 77 | 0.03548 |
| 78 | 0.03967 |
| 79 | 0.04527 |
| 80 | 0.05061 |
| 81 | 0.05651 |
| 82 | 0.06412 |
| 83 | 0.07130 |
| 84 | 0.07768 |
| 85 | 0.08708 |

Table S3. The result of subgroup analysis.

| Subgroup | Estimated OS HR | Estimated PFS HR | Incremental Cost, $ | Incremental QALYs | ICER, $/QALY |
| --- | --- | --- | --- | --- | --- |
| Tumor PD-L1 expression ≥1% | 0.75 | 0.60 | 83,219.66 | 0.85 | 97,905.48 |

Abbreviations: OS, overall survival; PFS, progression-free survival; HR, hazard ratio; QALY, quality-adjusted life-year; ICER, incremental cost-effectiveness ratio.

Table S4. ICER at Different Cost Reductions for Nivolumab.

| Cost of Cost of Nivolumab, % | Icer |
| --- | --- |
| 0.5 | 68440.62 |
| 0.6 | 54723.01 |
| 0.7 | 41005.39 |
| 0.721 | 38124.69 |
| 0.8 | 27287.78 |
